# Atypical Cutaneous Viral Infections Reveal an Inborn Error of Immunity in 8 Patients

**DOI:** 10.3390/microorganisms11051202

**Published:** 2023-05-04

**Authors:** Assiya El Kettani, Fatima Ailal, Farida Marnissi, Fouzia Hali, Jalila El Bakkouri, Ibtihal Benhsaien, Tom Le Voyer, Mame Sokhna Guèye, Rémi Chevalier, Soumiya Chiheb, Khalid Zerouali, Emmanuelle Jouanguy, Jean-Laurent Casanova, Ahmed Aziz Bousfiha

**Affiliations:** 1 Laboratory of Clinical Immunology-Inflammation and Allergy LICIA, Faculty of Medicine and Pharmacy, Hassan II University, Casablanca 20250, Morocco; 2 Laboratory of Bacteriology, Virology and Hospital Hygiene, Ibn Rochd University Hospital, Casablanca 20250, Morocco; 3 Laboratory of Bacteriology and Virology, Faculty of Medicine and Pharmacy, Hassan II University, Casablanca 20250, Morocco; 4 Clinical Immunology and Infectious Pediatrics Department, Abderrahim Harouchi Hospital, Ibn Rochd University Hospital, Casablanca 20250, Morocco; 5 Laboratory of Pathological Anatomy, Ibn Rochd University Hospital, Hassan II University, Casablanca 20250, Morocco; 6 Department of Dermatology, Ibn Rochd University Hospital, Hassan II University, Casablanca 20250, Morocco; 7 Immunology Laboratory, Ibn Rochd University Hospital, Casablanca 20250, Morocco; 8 Laboratory of Human Genetics of Infectious Diseases, Necker Branch, Institut National de la Santé et de la Recherche Médicale (INSERM), 75015 Paris, France; 9 Imagine Institute, Paris Cité University, 75015 Paris, France; 10 Institute for Health Research, Epidemiological Surveillance and Training, Dakar 7325, Senegal; 11 Laboratory of Human Genetics of Infectious Diseases, Rockefeller Branch, Rockefeller University, New York, NY 10065, USA; 12 Howard Hughes Medical Institute, Chevy Chase, MD 20815, USA

**Keywords:** viral infection, skin infection, inborn errors of immunity, diagnosis

## Abstract

Unusual viral skin infections might be the first clinical manifestation in children with an inborn error of immunity (IEI). We performed a prospective study from 1 October 2017 to 30 September 2021, at the Department of Pediatric Infectious Diseases and Clinical Immunity of Ibn Rochd University Hospital-Casablanca. During this period, on 591 patients newly diagnosed with a probable IEI, eight of them (1.3%), from six independent families, had isolated or syndromic unusual viral skin infections, which were either profuse, chronic or recurrent infections, and resistant to any treatment. The median age of disease onset was nine years old and all patients were born from a first-degree consanguineous marriage. By combining clinical, immunological and genetic investigations, we identified GATA2 deficiency in one patient with recalcitrant profuse verrucous lesions and monocytopenia (1/8) and STK4 deficiency in two families with HPV lesions, either flat or common warts, and lymphopenia (2/8), as previously reported. We also identified COPA deficiency in twin sisters with chronic profuse Molluscum contagiosum lesions, pulmonary diseases and microcytic hypochromic anemia (2/8). Finally, we also found one patient with chronic profuse MC lesions and hyper IgE syndrome, (1/8) and two patients with either recalcitrant profuse verrucous lesions or recurrent post-herpetic erythema multiforme and a combined immunodeficiency (2/8) with no genetic defect identified yet. Raising clinicians awareness that infectious skin diseases might be the consequence of an inborn error of immunity would allow for optimized diagnosis, prevention and treatment of patients and their families.

## 1. Introduction

Inborn errors of immunity (IEI) refer to a heterogeneous group of rare genetic disorders characterized by the lack or an impaired function of one or more components of the immune system. To date, 450 different IEIs have been identified, which affect either the adaptive, the innate, or both, immune responses [1]. Susceptibility to infectious diseases is a clinical phenotype commonly shared by patients with IEI. However, IEIs are usually underdiagnosed in patients with cutaneous viral infections, mainly because these clinical manifestations are not life-threatening and often isolated, whereas they might reveal these rare conditions [2].

Different families of viruses present a restrictive, or not, skin tropism and could infect the stratified epithelia of the skin. Cutaneous infections with viruses are common in the general population, usually benign, and spontaneously resolving. However, in few individuals otherwise healthy, they become severe due to chronicity, profusion, high recurrency, and/or resistance to any treatment. This should suggest a primary or acquired immune deficiency, underlying susceptibility to these infections. Most frequent viral infections reported in patients with IEI are alpha herpes virus (herpes simplex, varicella/zoster), molluscum contagiosum (MC) and human papillomavirus (HPVs) [3,4]. The nature of the lesions is depending of the virus involved. The lesions due to Herpes infections are painful erythematous papules, vesicles and shallow ulcers [4]. MC lesions are characterized by discrete, pearly white to pink, dome-shaped, umbilicated papules. Lesions often occur in clusters or a linear distribution due to autoinoculation [5]. HPV-induced lesions have a large clinical phenotype spectrum from epidermodysplasia verruciformis (EV), a rare disease due to β-HPV and characterized by flat warts, to profuse, persistent and recalcitrant common warts, due to α-, γ-, and μ-genera [4,6].

The genetic investigation of patients with atypical viral skin infections led to the identification of many IEIs. Patients with EV, either isolated or syndromic, carry mutations affecting either the EVER1-EVER2-CIB1 complex, which is acting as a restriction factor to HPVs in keratinocytes [7], or the number or the function of T cells, such as RHOH and STK4, both associated with T cell lymphopenia [6,7]. In contrast, most of the IEIs related to either an isolated or syndromic susceptibility to infections, including severe cutaneous viral diseases, are affecting, at least, the T cells compartment, either quantitatively (lack or decreased numbers of some subsets; e.g., CXCR4, GATA2), qualitatively (normal counts but an impaired function of T cells; e.g., CARMIL2) or both (e.g., DOCK8) [2,8]. Altogether, the study of patients with atypical viral skin infections showed the important role of T cells in the skin immune response against most of viruses involved and the necessary cooperation between skin-resident cells and immune cells.

However, the clinical penetrance of skin viral diseases varies a lot depending of the IEI from, e.g., 100% of EV phenotype in EVER1- or EVER2-deficient patients to 80 and 40% of HPV-induced warts in patients with CXCR4, and DOCK8 deficiencies, respectively [9,10]. The report of additional patients will help to better define the infectious susceptibility related to a specific IEI and to better dissect the immune response involved. In this study, we describe a series of patients with atypical cutaneous viral infections. Their clinical and immunological features suggested an IEI, which has been identified for some of them.

## 2. Material and Methods

This is a prospective longitudinal study that took place from 1 October 2017 to 30 September 2021, at the Department of Pediatric Infectious Diseases and Clinical Immunity of the Ibn Rochd University Hospital of Casablanca. Informed consent has been obtained from patients or their tutors when they are minors.

All patients seen in the dermatology or pediatrics consultation at Ibn Rochd University hospital were evaluated and recruited with the following criteria:

A- Clinical: patients with atypical isolated or syndromic cutaneous viral infections. To be classified as atypical, these infections have to be either chronic or recurrent, profuse (more than 20 lesions in more than one region of the body for warts) or recalcitrant and resistant to treatment (6 months for warts) [7].
B- Immunological: patients with abnormal blood cell, lymphocytes subpopulations or immunoglobulins count, in the absence of any recent immunosuppressive treatment or HIV infection.

An immune deficiency assessment was performed in these patients: Blood was collected on BD Vacutainer tube coagulation activator for HIV serology with the ARCHITECT^®^ HIV Ag/Ab Combo assay, Blood was collected on BD Vacutainer EDTA Tube for blood cell count on XN-3000 Sysmex^®^, lymphocyte subpopulation count with flow cytometry on BD Facscanto II ^®^, and immunoglobulin assay on SPA plus ^®^. Molecular biology tests: PCR on Bio-Rad T100 thermal cycler in search of candidate genes before sequencing. Sequence analysis and deletion/duplication testing of the 407 genes listed in the Genes Analyzed section of a custom-designed Illumina SNP array or by whole-exome sequencing was done. All targeted regions were sequenced with ≥50× depth. Reads were aligned to a reference sequence (GRCh37), and sequence changes were identified and interpreted in the context of a single clinically relevant transcript [11]. Sanger sequencing was performed to confirm the identified variant.

Anatomopathological assessment on punch biopsies of verrucous lesions was done too. The biopsies were fixed in formalin, dehydrated, fully embedded in paraffin, cut with a microtome then deparaffinized and stained with hematoxylin eosin, as described previously [12].

The diagnosis of IEI has been established according to the criteria defined by the International Union of Expert Committees of Immunology and Primary Immunodeficiency Societies [13].

## 3. Results

**Figure 1:**
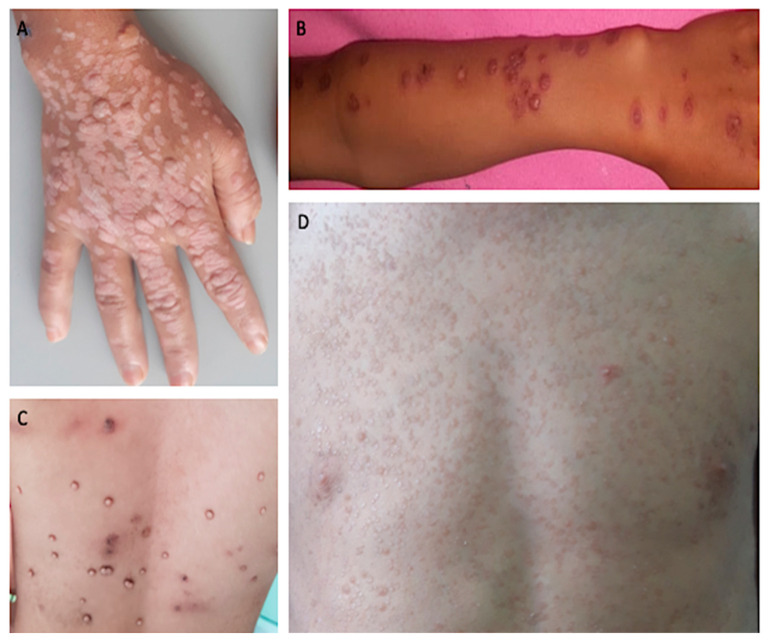
Clinical presentation of severe cutaneous viral infections. (**A**): Flat and vulgar warts on the right hand (Patient 2). (**B**): Cocade lesions on the forearm of the patient with postherpetic erythema multiforme (Patient 3). (**C**): Molluscun contagiosum lesions on the back (Patient 4). (**D**): Epidermodysplasia verruciformis lesions on the chest (Patient 1).

During the study period, 591 patients were diagnosed with a probably IEI and among them, eight patients, from six unrelated families met the study inclusion criteria (1.3%). Among them, 6 were females and 2 males. They ranged in age from 7 to 26 years with a median age of disease onset at 9 years old. Of these patients, 4 presented with recalcitrant profuse warts, 3 had chronic profuse lesions of Molluscum contagiosum, and one had recurrent postherpetic erythema multiforme (3rd episode in the current year) (Figure 1). In addition of their skin viral manifestations, some of the patients developed other symptoms (Table 1). To summarize, 5 had also pulmonary infections, 2 had digestive manifestations. In one family with two patients, sarcoidosis was diagnosed. Finally, in the twin sisters, microcytic hypochromic anemia was also diagnosed. None of the parents and siblings developed any clinical manifestations, except in one family in which a sister had diarrhea but no skin disease (Table 1).

**Figure 2:**
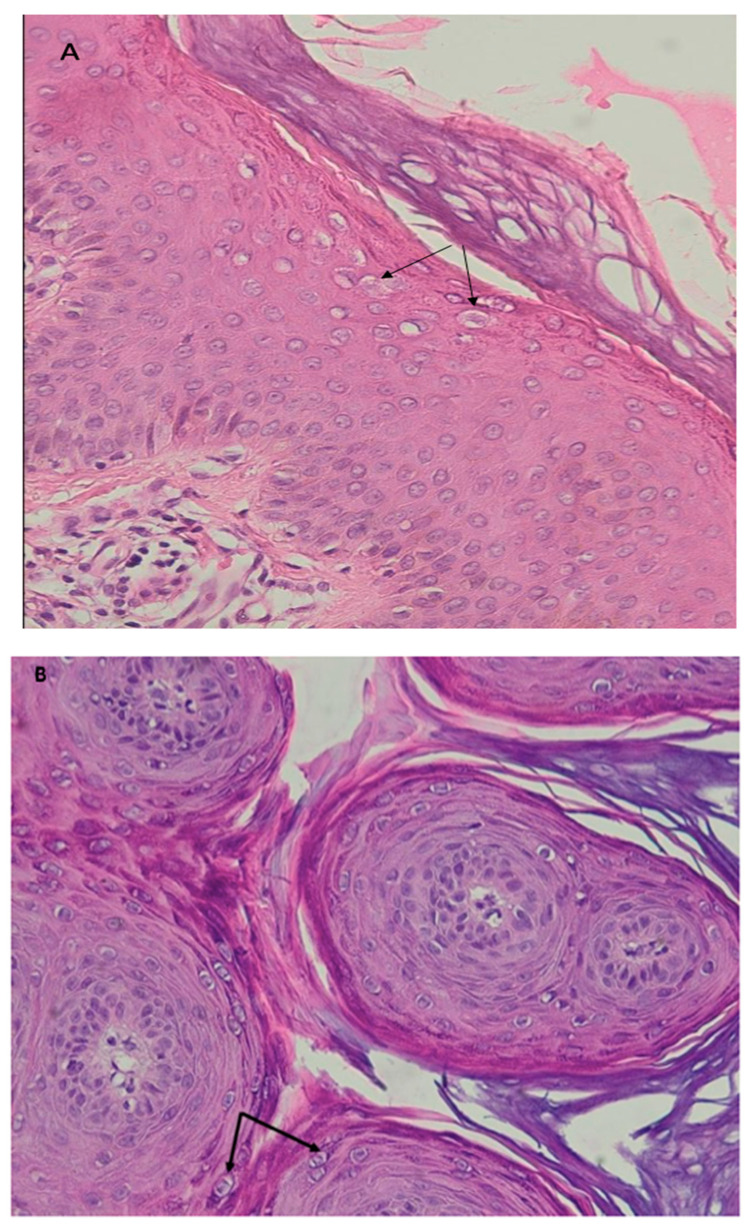

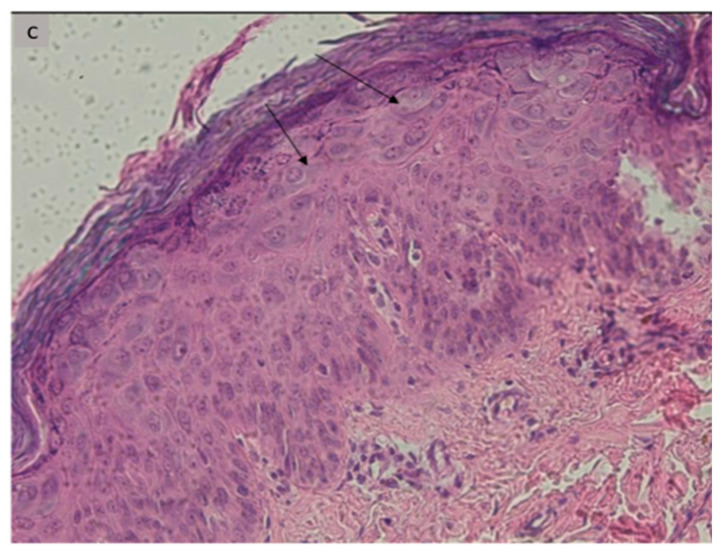
Hematoxylin and eosin (H&E) staining of HPV-induced lesions (**A**): Histological analysis showed koilocytes in the upper epidermidis of a flat wart (arrow) (×20, Patient 2)) (**B**): Koilocytes in the upper epidermidis of a vulgar wart (arrow) (×20, Patient 7). (**C**): Keratinocytes (arrow) with pale cytoplasm epidermidis in epidermodyplasia verruciformis lesion (×40, Patient 1).

To pursue the clinical characterization, we performed histological staining. The flat and vulgar warts were unremarkable in these patients. Flat warts, showed orthokeratosis, mild papillomatosis, and mild acanthosis, with no or minimal parakeratosis. The dermis was normal. The upper epidermis had hypergranulosis and numerous koilocytes (Figure 2A). Vulgar warts showed markedly papillomatous epidermis with hypergranulomatosis and overlying parakeratosis. The upper epidermis contained large pink inclusions, particularly in cases arising on acral skin. Other lesions showed smaller basophilic granules. In the upper epidermis, koilocytes or vacuolated keratinocytes which have a small shrunken nucleus surrounded by a perinuclear halo were observed (Figure 2B). As expected, the EV lesions presented with hyperkeratosis and parakeratosis, mild acanthosis and the presence of koilocytes, keratinocytes with pale cytoplasm in the upper epidermis associated with high levels of intranuclear viral replication. The cytoplasm of the affected cells was stained pale blue with no evidence of malignancy (Figure 2C) [14].

At the immunological level, we observed that most of the patients had either a decreased number of leukocytes (5/8), a lymphopenia (4/8), a neutropenia (1/8) or in contrast an increased number of polynuclear eosinophils and hyper IgE (1/8). T cell subsets were evaluated for some patients and we observed that both CD4^+^ and CD8^+^ T cells were affected (Table 1). Genetic investigations allowed us to identify predicted loss-of-function (pLOF) variants in STK4, a missense variant in GATA2, and a missense variant in COPA (Table 1). Further investigations might be necessary to validate the functional impact. As for the patient with recurrent post herpetic erythema multiforme and the patient with a LOCID and recalcitrant profuse vulgar warty lesions, lymphopenia was profound but there was no genetic defect detected according to the panel used for the search of inborn errors of immunity. For all patients without genetic etiology yet, the whole exome sequencing is needed to complete our investigations.

## 4. Discussion

We report here a series of eight patients with isolated or syndromic severe viral skin diseases. This initial clinical observation led us to perform immunological and genetic investigations, which allowed us the do a clinical diagnosis for all patients based on immunological results, such as hyper IgE, and a genetic diagnosis for most of them. Severe skin infections are a common clinical phenotype shared by several groups of inborn errors of immunity [1], as illustrated in our series. We have found one family with Molluscum lesions, pulmonary diseases and COPA deficiency; one patient with HPV lesions, monocytopenia and GATA2 deficiency and two families with HPV lesions, either EV or common warts, lymphopenia and STK4 deficiency. At the immunological level, all these IEI share to impact the T lymphocytes, either quantitatively or qualitatively.

Patients with STK4 and GATA2 deficiencies, reported in this series, have similar clinical and immunological phenotypes than the previous ones: susceptibility to HPVs, T cells lymphopenia and monocytopenia, respectively [6]. Indeed, AR mutations in STK4 gene are primarily characterized by a reduced amount and survival of circulating naïve T cells. Progressive CD4 T cell lymphopenia with profoundly low naïve CD4 T cell counts is hallmark, while CD8 T cells and NK cells are within normal range. T cell proliferation responses to both antigens and mitogens are markedly impaired. B cell counts are mildly low with hypergammaglobulinemia of IgG and variable increases in IgA and IgE [15]. Furthermore, in GATA2 haploinsufficiency, α-HPV infections occur in more than 50% of the cases, and HPV-induced genital cancers are frequent. Low monocyte, DC, B cell, CD4^+^ T cell, and NK cell counts are the most common immunological features of the patients [8]. COPA syndrome is associated with autoantibody development, increased Th17 cells and pro-inflammatory cytokine expression including IL-1β and IL-6 [16].

In the particular case of EV, it has been shown that keratinocytes and T cells are essential to control β-HPV. Indeed, patients with EVER1, EVER2 or CIB1 deficiencies developed only isolated EV. They may develop non carcinoma skin cancer after decades [17]. None of the patients displayed a T cell deficiency, suggesting that the keratinocyte might be the defective cell. In contrast, patients with syndromic, or atypical, EV have always a T cell deficiency, as patients with non-EV HPV-induced lesions [17]. Indeed, T lymphocytes are key cells in the control of skin diseases, following herpes viruses, MC or HPVs infections. In autosomal recessive (AR) DOCK8 deficiency, warts were reported in >40% of patients that were characterized by T and NK cell lymphopenia [10]. In autosomal dominant (AD) CXCR4, almost 80% of the patients developed HPV-induced lesions [9] The high prevalence of HPV manifestations might be the consequence of the defective migration of T cells (DOCK8), of the lack of interaction between antigen-presenting cells and T cells (GATA2, CXCR4), an impaired of T cell activation (CARMIL2, CD28). However, to be able to dissect the antiviral skin immune response, it is essential to diagnose first at the clinical level and then at the immunological and genetic levels all the patients concerned. [9].

## 5. Conclusions

Unusual cutaneous viral infections might be the consequence of an inborn error of immunity. It is essential to sensibilize the physicians and anatomo-pathologists regarding this particular pathological field of primary immunodeficiencies, to optimize the delay of clinical and immunological diagnosis, as well as the knowledge of the genetic basis of these disorders will guarantee an early diagnosis and optimized treatment of the patients and their families.

## Figures and Tables

**Table 1:** Clinical and paraclinical characteristics of patients with atypical cutaneous viral infections.

Pt	Age of Onset (yrs)	Sex	Reason for Consultation	Site of the Lesions	Family and Personal History	Blood Cell Count Unit: cells/mm^3^ (Pathological)	Lymphocyte/Immunoglobulin Subpopulations (Pathological)	Histology	Genetic Diagnosis (Mutation)	Diagnosis
1	12	M	Recalcitrant profuse verrucous lesions (flat and pityriasiform warts) since the age of 6 years	Face, neck, trunk, arms	Sister followed for CVID Recurrent diarrhea since the age of 10	Lymphopenia 900/mm^3^	CD3^+^: 771/mm^3^ CD4^+^: 253/mm^3^ CD19^+^: 128/mm^3^ CD16^+^, CD56^+^: 743/mm^3^	Koilocytes, keratinocytes with pale blue cytoplasm in the upper epidermis associated with high levels of intranuclear viral replication	STK4 (homozygous, c.1305 + 1G > A)	LOCID+ Syndromic epidermo- dysplasia verruciformis
2	26	F	Recalcitrant profuse verrucous lesions (flat and vulgar warts) since the age of 15 years	Face, forearms, hands, legs, feet	No special features	Monocytopenia 100/mm^3^	CD19^+^: 9/mm^3^ CD16^+^, CD56^+^: 64/mm^3^ Absence of CD14^+^ monocytic cells	Papillomatous epidermis related to common warts	GATA2 (c.1070C > T, p.T357I)	DCML deficiency
3	10	M	Recurrent post-herpetic erythema multiforme (cocade lesions) since the age of 9	Face, arms, forearms, hands	Two identical episodes of post-herpetic erythema multiforme in the current year	Lymphopenia 1050/mm^3^	CD3^+^: 945/mm^3^ CD4^+^: 453/mm^3^ CD8^+^: 338/mm^3^	-	-	CID
4	9	F	Profuse chronic Molluscum contagiosum since the age of 2	Face, trunk, arms, forearms, hands	Twin sister of patient 5 Since the age of one: Recurrent respiratory infections At 2 years old: sarcoidosis	Microcytic hypochromic anemia	Normal	-	COPA (c.3653T > C, p.Ile1218Thr)	CID
5	9	F	Profuse chronic Molluscum contagiosum since the age of 2	Face, trunk, arms, forearms, hands	Twin sister of patient 4 Since the age of one: Recurrent respiratory infections At 2 years old: sarcoidosis	Microcytic hypochromic anemia	Normal	-	COPA (c.3653T > C p.Ile1218Thr)	CID
6	8	F	Profuse recalcitrant warty lesions (flat and pityriasiform warts) Since the age of 3 years	Face, neck trunk, arms, forearms, hands	Recurrent respiratory infections since the age of 3 years, with stature weight repercussions, Diarrhea	Neutropenia 580/mm^3^ Lymphopenia: 1750/mm3	CD4^+^: 254/mm^3^ CD19^+^:130/mm^3^ CD16^+^, CD56^+^: 64/mm^3^	Koilocytes, keratinocytes with pale blue cytoplasm in the upper epidermis associated with high levels of intranuclear viral replication	STK4 (homozygous, c.750G > A, p.W250)	CID+ Syndromic epidermo- dysplasia verruciformis
7	18	F	Recalcitrant profuse warty lesions (flat and vulgar warts) since the age of 10 years	Face, neck, trunk, arms, forearms, hands, thighs, legs, feet Recurrent diarrhea	Recurrent respiratory infections	Leukocytes: 3160/mm^3^ lymphopenia: 430/mm^3^	CD4^+^: 171/mm^3^ CD8^+^: 182/mm^3^ CD19^+^: 22/mm^3^ CD16^+^, CD56^+^: 90/mm^3^	Papillomatous epidermis related to common warts with no particularity	-	LOCID
8	7	F	Profuse chronic Molluscum contagiosum lesions	Trunk, arms, forearms	For the past year and a half: -Recurrent respiratory infections -leukorrhea, vulvitis -Eczema lesions	High PNE: 1070/mm^3^	IgE: 162 kUI/l	-	-	Hyper IgE syndrome

STK4: serine-threonine protein kinase 4, GATA2: GATA-binding protein 2, COPA: COPI coat complex Subunit alpha. Yrs: years; DCML, dendritic cell, monocyte, B cell and NK cell lymphopenia, LOCID: late onset combined immunodeficiency, CID: combined immunodeficiency, PNE: polynuclear eosinophils.

## Data Availability

Not applicable.

## References

[1] Bousfiha A., Jeddane L., Picard C., Al-Herz W., Ailal F., Chatila T., Cunningham-Rundles C., Etzioni A., Franco J.L., Holland S.M. (2020). Human Inborn Errors of Immunity: 2019 Update of the IUIS Phenotypical Classification. J. Clin. Immunol..

[2] Saeidian A.H., Youssefian L., Huang C.Y., Palizban F., Naji M., Saffarian Z., Mahmoudi H., Goodarzi A., Sotoudeh S., Vahidnezhad F. (2022). Whole-transcriptome sequencing–based concomitant detection of viral and human genetic determinants of cutaneous lesions. J. Clin. Investig..

[3] Orth G. Les papillomavirus, “du col à la peau”.

[4] Beauman J.G. (2005). Genital herpes: A review. Am. Fam. Physician.

[5] Brown J., Janniger C.K., Schwartz R.A., Silverberg N.B. (2006). Childhood molluscum contagiosum. Int. J. Dermatol..

[6] El Kettani A., Ailal F., El Bakkouri J., Zerouali K., Béziat V., Jouanguy E., Casanova J.L., Bousfiha A.A. (2022). HPV-Related Skin Phenotypes in Patients with Inborn Errors of Immunity. Pathogens.

[7] de Jong S.J., Créquer A., Matos I., Hum D., Gunasekharan V., Lorenzo L., Jabot-Hanin F., Imahorn E., Arias A.A., Vahidnezhad H. (2018). The human CIB1-EVER1-EVER2 complex governs keratinocyte-intrinsic immunity to β-papillomaviruses. J. Exp. Med..

[8] Béziat V., Jouanguy E. (2021). Human inborn errors of immunity to oncogenic viruses. Curr. Opin. Immunol..

[9] Béziat V. (2020). Human genetic dissection of papillomavirus-driven diseases: New insight into their pathogenesis. Hum. Genet..

[10] Aydin S.E., Kilic S.S., Aytekin C., Kumar A., Porras O., Kainulainen L., Kostyuchenko L., Genel F., Kütükcüler N., Karaca N. (2015). DOCK8 Deficiency: Clinical and Immunological Phenotype and Treatment Options—A Review of 136 Patients. J. Clin. Immunol..

[11] Genetic Test Catalog: Genetic Test Panels from Invitae. https://www.invitae.com/en/providers/test-catalog.

[12] Davy C., Doorbar J. (2005). Human Papillomaviruses: Methods and Protocols.

[13] Picard C., Bobby Gaspar H., Al-Herz W., Bousfiha A., Casanova J.L., Chatila T., Crow Y.J., Cunningham-Rundles C., Etzioni A., Franco J.L. (2018). International Union of Immunological Societies: 2017 Primary Immunodeficiency Diseases Committee Report on Inborn Errors of Immunity. J. Clin. Immunol..

[14] Orth G. (2006). Genetics of epidermodysplasia verruciformis: Insights into host defense against papillomaviruses. Semin. Immunol..

[15] Abdollahpour H., Appaswamy G., Kotlarz D., Diestelhorst J., Beier R., Schäffer A.A., Gertz E.M., Schambach A., Kreipe H.H., Pfeifer D. (2012). The phenotype of human STK4 deficiency. Blood.

[16] Vece T.J., Watkin L.B., Nicholas S.K., Canter D., Braun M.C., Guillerman R.P., Eldin K.W., Bertolet G., McKinley S.D., de Guzman M. (2016). Copa Syndrome: A Novel Autosomal Dominant Immune Dysregulatory Disease. J. Clin. Immunol..

[17] de Jong S.J., Imahorn E., Itin P., Uitto J., Orth G., Jouanguy E., Casanova J.L., Burger B. (2018). Epidermodysplasia Verruciformis: Inborn Errors of Immunity to Human Beta-Papillomaviruses. Front. Microbiol..

